# 0976. Similar effect of albumin given either as a rapid bolus or slow infusion in a large animal model of sepsis

**DOI:** 10.1186/2197-425X-2-S1-P68

**Published:** 2014-09-26

**Authors:** M von Seth, P Engström, A Larsson, L Hillered, E Maripuu, C Widström, M Lipcsey, J Sjölin

**Affiliations:** Surgical Sciences, Uppsala Universitet, Uppsala, Sweden; Department of Anaesthesia, Hudiksvall Hospital, Hudiksvall, Sweden; Medical Sciences, Uppsala University, Uppsala, Sweden; Neurosciences, Uppsala University, Uppsala, Sweden; Medical Physics, Akademiska Ssjukhuset, Uppsala, Sweden; Surgical Sciences, Uppsala University, Uppsala, Sweden

## Introduction

The strategy of fluid resuscitation in severe sepsis and septic shock is a matter of ever-ongoing debate. Current sepsis guidelines recommend albumin in patients requiring large amounts of crystalloids for circulatory stability. Although the same guidelines also recommend bolus administration of fluid, concerns have been raised that fluid boluses might lead to increased extravasation of albumin leading to sustained tissue edema in patients with sepsis-induced activation of the systemic inflammatory response.

## Objectives

We hypothesized that a slow infusion of 10 mL x kg^-1^ 5% albumin would lead to less capillary leakage of the given albumin compared to the same amount given as a rapid bolus. This can be studied in a porcine intensive care model of sepsis-induced systemic inflammatory response.

## Methods

The systemic inflammatory response was induced by endotoxin in a dose of 1 µg x kg^-1^ x h^-1^ resulting in severe sepsis and septic shock in all animals. Thirty-two animals were monitored and ventilated with standard intensive care equipment and randomized to either a 2-hour continuous infusion (INF group; n=16) or a rapid bolus (BOLUS group; n=16) of 10 mL x kg^-1^ of 5% albumin labeled with Technetium^99m^. Blood hemoglobin levels were used as a measure of systemic inflammatory response-induced capillary leakage. Radioactivity was monitored in plasma, urine and muscle microdialysate for a total of 6 hours. Post mortem analyses of radioactivity in liver, spleen, kidney and lung were performed and total lung water was assesed.

## Results

The two groups were similar at baseline. Hemoglobin increased in both the INF and the BOLUS groups with no differences between the two (19±8 vs. 19±9 gxL^-1^; mean±SD, p< 0.001 for both). Radioactivity in plasma differed between the two groups during the infusion, corresponding to the pattern of albumin administration (Figure 1). During the 3 hours following the fluid administration, there were no differences in the rate of decrease in plasma radioactivity between the INF and BOLUS groups (-3.1x10^5^±7.2x10^4^ vs. -3.2x10^5^±6.4x10^4^; mean±SD, n.s.). There were no differences between groups in muscle microdialysate/blood radioactivity ratio at 6 hours (1.9 [0.6-26] vs. 2.7 [1.2-4.2] %; median [IQR]; n.s.; INF and BOLUS groups, respectively). No intergroup differences were seen in the urine radioactivity during the study period or in lung dry/wet ratio or radioactivity of viscera at 6 h. Finally, there were no differences between the two groups in hemodynamics, respiration, hematology or renal function during the experiment.

## Conclusions

In an intensive care large animal model of sepsis, administration of significant volume of 5% albumin as rapid bolus compared to slow infusion did not lead to greater extravasation of albumin.

